# Clinicopathological factors affecting the effect of neoadjuvant chemotherapy in patients with gastric cancer

**DOI:** 10.1186/s12957-021-02157-x

**Published:** 2021-02-09

**Authors:** Lin Jiang, Zhiqiang Ma, Xin Ye, Weiming Kang, Jianchun Yu

**Affiliations:** 1 Department of General Surgery, Peking Union Medical College Hospital, Chinese Academy of Medical Sciences and Peking Union Medical College, No. 1 Shuaifuyuan, Wangfujin, Dongcheng District, Beijing, 100730 China; 2 Graduate School, Chinese Academy of Medical Sciences and Peking Union Medical College, Beijing, 100005 China

**Keywords:** Gastric cancer, Neoadjuvant chemotherapy, Pathological response, Clinicopathological factors, Chemotherapy effect

## Abstract

**Background:**

Neoadjuvant chemotherapy is an important part of the comprehensive treatment of advanced gastric cancer (GC). The effect of neoadjuvant chemotherapy plays a key role in the prognosis of GC patients. Pathological response can represent the effect of neoadjuvant chemotherapy. However, evidence focused on pathological response and associated clinicopathological factors in GC patients is quite little. In this retrospective study, the clinicopathological factors affecting the effect of neoadjuvant chemotherapy in GC patients were investigated, and suggestions were proposed to improve the effect of neoadjuvant chemotherapy on GC.

**Methods:**

Retrospective analysis was performed on GC patients who received radical surgery after neoadjuvant chemotherapy from February 2016 to December 2019 at Peking Union Medical College Hospital. Relevant clinicopathological data was collected to analyze the factors influencing the effect of neoadjuvant chemotherapy. Chi-square test was used for univariate analysis. Logistic regression was used for multivariate analysis. Receiver operating characteristic curve (ROC) was used to determine the cutoff value of variables which significantly influenced the effect of neoadjuvant chemotherapy.

**Results:**

A total of 203 GC patients were included in the study. Analyses showed that patients < 60 years old (OR = 1.840 [1.016–3.332], *P* = 0.044), histological type of poor differentiation or signet-ring cell carcinoma (OR = 2.606 [1.321–5.140], *P* = 0.006), and weight loss during neoadjuvant chemotherapy (OR = 2.110 [1.161–3.834], *P* = 0.014) were independent risk factors for neoadjuvant chemotherapy effect. In ROC analysis of weight change and neoadjuvant chemotherapy effect, area under the curve (AUC) was 0.593 (*P* = 0.024) and cutoff value of weight change was − 2.95%. Chi-square test showed that patients without weight loss during neoadjuvant chemotherapy had a higher rate of oral nutritional supplement (ONS) than patients with weight loss (*P* = 0.039).

**Conclusions:**

Patients <60 years old, histological type of poor differentiation or signet-ring cell carcinoma, and weight loss during neoadjuvant chemotherapy were independent risk factors for neoadjuvant chemotherapy effect in GC patients. Patients with weight loss > 2.95% during neoadjuvant may have a worse chemotherapy effect. Timely nutritional support such as ONS to maintain patients’ body weight is crucial for improving the effect of neoadjuvant chemotherapy.

## Background

Gastric cancer (GC) is a common malignant tumor in the world with a poor prognosis and a serious threat to human health. According to statistics from the International Agency for Research on Cancer (IARC) and the World Health Organization (WHO), in 2018, there were about 1.034 million new cases of GC worldwide, and 783,000 deaths due to GC, ranking 6th in the incidence and 3rd in the mortality of malignant tumors [1]. According to the latest data revealed by the National Cancer Registration Center of China, in 2015, there were about 679,000 new cases and 498,000 deaths of GC in China; the morbidity and mortality of GC ranked 2nd in China only behind lung cancer [2]. The increasing burden of seeking resolutions for prevention and treatment of GC is receiving attention worldwide.

Currently, the combination of neoadjuvant chemotherapy, surgery, and adjuvant chemotherapy is an important mode of GC treatment globally. A number of studies showed that compared to surgery alone, this mode was beneficial to tumor downstaging, improving the rates of R0 resection, prolonging the patients’ survival, and the patient’s postoperative complications did not increase [3–5]. However, according to the latest statistics, the 5-year survival rate of GC in China is still at a low level, only 35.9% [6]. Besides low early diagnosis rate and high proportion of advanced GC, chemotherapy resistance is another critical cause. The development of drug resistance greatly limits the efficacy of chemotherapy and ultimately leads to chemotherapy failure, tumor progression, or recurrence [7, 8]. Therefore, revealing the mechanism of chemotherapy resistance and improving the sensitivity of chemotherapy have become a hotspot in the field of GC research.

Pathological response can represent the effect of neoadjuvant chemotherapy. Good pathological response means good effect of neoadjuvant chemotherapy [9, 10]. Therefore, improving pathological response can improve the effect of neoadjuvant chemotherapy. Based on our clinical experience, we found that several relevant clinicopathological factors may influence the pathological response. For example, patients with low body weight generally had poor chemotherapy tolerance and poor pathological response. However, evidence focused on pathological response and associated clinicopathological factors in GC patients is quite little. Accordingly, the aim of our study was to find out which clinicopathological factors can influence the pathological response of neoadjuvant chemotherapy in GC patients at a medical center.

## Methods

### Patients and study design

GC patients who received radical surgery after neoadjuvant chemotherapy from February 2016 to December 2019 at Peking Union Medical College Hospital were screened for inclusion. The inclusion criteria were as follows: (1) Patients were diagnosed as gastric adenocarcinoma by endoscopic biopsy; (2) Patients were evaluated as advanced GC by imaging examination, mainly by contrast-enhanced computed tomography (CT) and endoscopic ultrasonography (EUS). The clinical stages were identified as T2 or T2+ (any N) according to the 8th edition American Joint Committee on Cancer (AJCC) Staging Manual [11]. (3) Patients received neoadjuvant chemotherapy first, and then received radical gastrectomy for cancer. (4) Postoperative pathological evaluation was complete including tumor regression grading. The exclusion criteria were as follows: (1) Patients were evaluated as early GC by imaging examination, mainly by contrast-enhanced CT and EUS. The clinical stages were identified as T1a or T1b (any N) according to the 8th edition AJCC Staging Manual. (2) Patients received radical gastrectomy for cancer directly without neoadjuvant chemotherapy. (3) Patients received chemotherapy or radiotherapy before the diagnosis of GC. (4) Patients were evaluated as late GC and lost the opportunity for radical surgery. Finally, 203 GC patients who received neoadjuvant chemotherapy followed by radical surgery were enrolled in the study. We further retrospectively collected the clinicopathological data of these patients to analyze the factors influencing the effect of neoadjuvant chemotherapy. This retrospective study was reviewed and approved by the Institutional Review Board of Peking Union Medical College Hospital. Each patient provided written informed consent.

### Histological type of biopsy

The diagnosis of GC depends on the biopsy of gastroscope. According to the Department of Pathology in our hospital, biopsy pathologies of GC were classified into four types: well-differentiated adenocarcinoma, moderately differentiated adenocarcinoma, poorly differentiated adenocarcinoma, and signet-ring cell carcinoma. Signet-ring cell carcinoma (SRCC) is a histological type based on more than 50% of the tumor containing abundant intracytoplasmic mucin pushing nucleus to the periphery, according to the WHO classification [12]. We regarded well-differentiated adenocarcinoma or moderately differentiated adenocarcinoma as low grade group and poorly differentiated adenocarcinoma or signet-ring cell carcinoma as high grade group in our study for comparison.

### Neoadjuvant chemotherapy regimen

According to the National Comprehensive Cancer Network (NCCN) and European Society for Medical Oncology (ESMO) guidelines, GC patients with clinical T stages T2 or T2+ are supposed to receive neoadjuvant chemotherapy regardless of the N stages [13, 14]. In the current study, our included patients’ clinical T stages were T2, T3, T4a, or T4b. Oxaliplatin plus S-1 (SOX) regimen was applied as the neoadjuvant chemotherapy regimen: S-1 was administered orally 80 mg/m^2^/day on days 1–14, while oxaliplatin was administered intravenously 130 mg/m^2^ on day 1. The treatment was repeated every 3 weeks. Since there were no clear criteria about how many courses of neoadjuvant chemotherapy should be performed before surgery, we usually performed 2–4 cycles for patients mainly according to their clinical T stages. More courses should be performed when the tumor stage was later. The interval between the last neoadjuvant chemotherapy and surgery was generally 1 month.

### Weight measurement

In our study, the body weights of the patients were recorded at two individual time points. We weighed the patients for the first time before starting neoadjuvant chemotherapy. Before the surgery but after the last neoadjuvant chemotherapy, we weighed the patients again. Patients with body weight declined during the neoadjuvant chemotherapy were classified as weight loss group. Patients with body weight maintained or increased during the neoadjuvant chemotherapy were classified as no weight loss group.

### Tumor regression grading

**Fig. 1:**
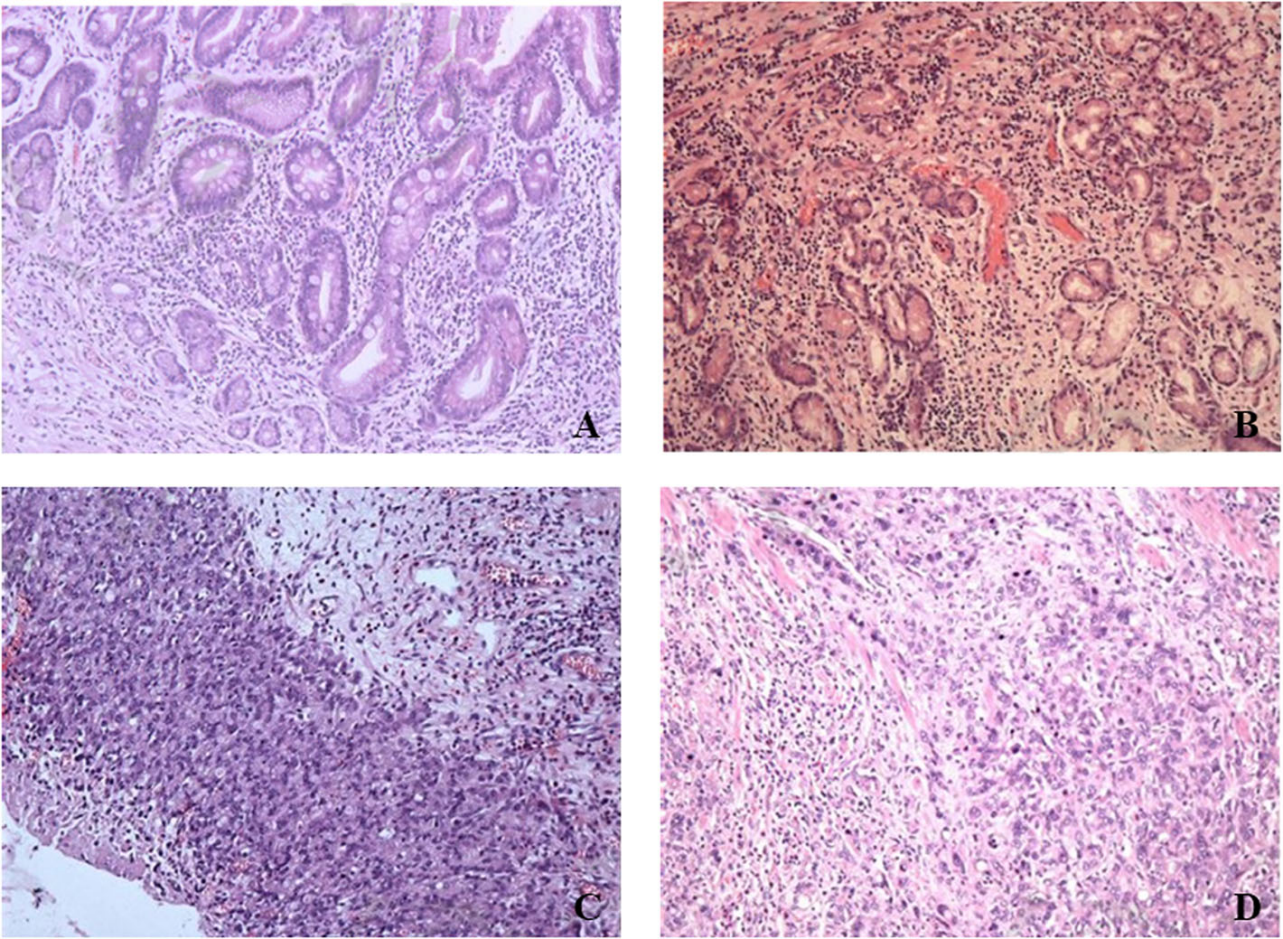
Histological images of CAP grading. **a** CAP 0, complete response to tumor treatment. Acute and chronic inflammation of the stomach wall with fibrous tissue hyperplasia. No viable cancer cells can be found. **b** CAP 1, almost complete response to tumor treatment. Residual adenocarcinoma in the submucosa of the gastric wall with extensive fibrous tissue hyperplasia. **c** CAP 2, partial response to tumor treatment. Local gastric cancer cells invade the extramuscular fat tissue with fibrous tissue hyperplasia. **d** CAP 3, no response to tumor treatment. Local gastric cancer cells invade the muscle layer with no evident tumor regression

In our study, the pathological response evaluation system of neoadjuvant chemotherapy referred to the College of American Pathologists (CAP) [15]. There are four grades in this tumor regression grading system: CAP 0 represents complete response: no viable cancer cells can be found; CAP 1 represents near complete response: single cells or rare small groups of cancer cells can be found. CAP 2 represents partial response: there is residual tumor with evident tumor regression; CAP 3 represents poor or no response: there is extensive residual tumor with no evident tumor regression (Fig. 1). In our study, CAP 0, CAP 1, and CAP 2 were defined as pathological response, which indicated good effect of neoadjuvant chemotherapy, while CAP 3 was defined as no pathological response, which indicated poor effect of neoadjuvant chemotherapy. To gain the CAP value, the pathological report of each patient was preliminarily written by one junior pathologist and then reviewed by another senior pathologist. Both of them were specialized in gastrointestinal diseases.

### Statistical analysis

Descriptive statistics of categorical variables focused on frequencies and proportions. Medians (ranges) were reported for continuous variables. The chi-square tests and multivariate logistic regression models tested the association between clinicopathological factors and pathological response to neoadjuvant chemotherapy. Receiver operating characteristic (ROC) curve analysis was used to figure out the cutoff value of variables which significantly influenced the effect of neoadjuvant chemotherapy. Statistical tests were performed using the Statistical Package for the Social Sciences (SPSS), version 23 (SPSS Inc., IBM Corp., Armonk, NY, USA). All tests were two sided, with a significance level set at 0.05.

## Results

### General condition of the patients

Two hundred and three GC patients who received neoadjuvant chemotherapy followed by radical surgery were enrolled in the study. Among the 203 patients, 155 were male while 48 were female. The median age of the patients was 63 years old (range, 26–81 years old). Among the 203 patients, according to the tumor regression grading from the postoperative pathology, 27 patients (13.3%) were CAP 0, 22 patients (10.8%) were CAP 1, 74 patients (36.5%) were CAP 2, and 80 patients (39.4%) were CAP 3 respectively. Based on our system, 123 (60.6%, CAP 0, 1, 2) patients had pathological response while 80 (39.4%, CAP 3) patients had no pathological response.

### Chi-square test for clinicopathological factors and pathological response

We first did the chi-square tests to find out the association between clinicopathological factors and pathological response to neoadjuvant chemotherapy (Table 1). Notably, age was the first critical factor we found as older patients (≥ 60 years old) showed a higher rate of pathological response than younger patients (66.4% vs 52.4%, *P* = 0.044). Histological type of biopsy was also related to pathological response as patients with well-differentiated or moderately differentiated adenocarcinoma had better pathological response than patients with poorly differentiated adenocarcinoma or signet-ring cell carcinoma (74.2% vs 54.6%, *P* = 0.009). There was a tendency that the higher clinical T (cT) stage of the patients, the poorer pathological response they had (*P* = 0.158). It also showed that patients without weight loss displayed a better but not significant trend for pathological response than patients with weight loss (66.4% vs 53.3%, *P* = 0.059). Additionally, sex (*P* = 0.977), smoking (*P* = 0.948), drinking (*P* = 0.669), clinical N stage (*P* = 0.745), tumor location (*P* = 0.204), and course of chemotherapy (*P* = 0.635) showed no significant influence on pathological response.

**Table 1:** Chi-square test for clinicopathological factors and pathological response after neoadjuvant chemotherapy in GC patients

Factors	No. of patients (total, *n* = 203)			χ^2^	*P*
	Total no. of rows	Pathological response (CAP 0, 1, 2)	No pathological response (CAP 3)		
**1. Age (years)**					
≥ 60	119	79	40	4.405	0.044
< 60	84	44	40		
**2. Sex**					
Male	155	94	61	0.001	0.977
Female	48	29	19		
**3. Smoking**					
Smokers	106	64	42	0.004	0.948
Non-smokers	97	59	38		
**4. Drinking**					
Drinkers	70	41	29	0.183	0.669
Non-drinkers	133	82	51		
**5. Smoking and drinking**					
Both	61	36	25	0.091	0.955
One of them	54	33	21		
Neither	88	54	34		
**6. cT stage**					
T2	21	16	5	5.192	0.158
T3	73	47	26		
T4a	101	57	44		
T4b	8	3	5		
**7. cN stage**					
N-	61	38	23	0.106	0.745
N+	142	85	57		
**8. Tumor location**					
Upper	47	34	13	4.589	0.204
Middle	65	40	25		
Lower	89	48	41		
Whole	2	1	1		
**9. Pathological type**					
Well-differentiated or moderately differentiated	62	46	16	6.917	0.009
Poorly differentiated or signet-ring cell	141	77	64		
**10. Course**					
2	39	23	16	0.908	0.635
3	57	32	25		
4	107	68	39		
**11. Weight change**					
No weight loss	113	75	38	3.567	0.059
Weight loss	90	48	42		

*GC* gastric cancer, *cT stage* clinical T stage, *cN stage* clinical N stage

### Multivariate analysis for clinicopathological factors and pathological response

Multivariate analysis was also performed in our study (Table 2). We selected the factors that differed significantly as well as the factors with a *P* value close to 0.1 in chi-square test. Finally, we enrolled age, clinical T stage, histological type of biopsy, and weight loss in the multivariate logistic regression analysis. After the statistical analysis, we found age, histological type of biopsy, and weight loss significantly influenced the pathological response. Patients ≥ 60 years old had better pathological response than patients < 60 years old (OR = 1.840, 95% CI 1.016–3.332, *P* = 0.044). Patients with well-differentiated or moderately differentiated adenocarcinoma showed better pathological response than patients with poorly differentiated adenocarcinoma or signet-ring cell carcinoma (OR = 2.606, 95% CI 1.321–5.140, *P* = 0.006). Patients without weight loss had better pathological response than patients with weight loss during neoadjuvant chemotherapy (OR = 2.110, 95% CI 1.161–3.834, *P* = 0.014). Age, histological type of biopsy, and weight loss were independent risk factors which influenced the effect of neoadjuvant chemotherapy.

**Table 2:** Multivariate analysis for clinicopathological factors and pathological response after neoadjuvant chemotherapy in GC patients

Factors	Odds ratio (95 % CI)	*P*
**1. Age (years)**		
≥ 60	1.840 (1.016–3.332)	0.044
< 60	1	
**2. cT stage**		
T2	1	
T3	0.592 (0.186–1.885)	0.375
T4a	0.419 (0.135–1.294)	0.131
T4b	0.197 (0.032–1.210)	0.079
**3. Pathological type**		
Well-differentiated or moderately differentiated	2.606 (1.321–5.140)	0.006
Poorly differentiated or signet-ring cell	1	
**4. Weight loss**		
No weight loss	2.110 (1.161–3.834)	0.014
Weight loss	1	

*GC* gastric cancer, c*T stage* clinical T stage, *CI* confidence interval

### Chi-square test to speculate why these three factors were risk factors

We conducted a chi-square test for age and histological type (Table 3) and found that the proportion of poorly differentiated adenocarcinoma or signet-ring cell carcinoma in older group (≥ 60 years old) and younger group (< 60 years old) was 68.1% and 71.4% respectively. There was no significant difference between the two groups (χ^2^ = 0.262, *P* = 0.609). Another chi-square test for weight loss and oral nutritional supplement (ONS) during neoadjuvant chemotherapy (Table 4) showed that patients without weight loss had a higher rate of ONS than patients with weight loss during neoadjuvant chemotherapy (82.3%% vs 70%, *χ*^2^ = 4.261, *P* = 0.039).

**Table 3:** Chi-square test for age and histological type

Age (years)	No. of patients (total, *n* = 203)			χ^2^	*P*
	Total no. of rows	Poorly differentiated or signet-ring cell	Well-differentiated or moderately differentiated		
**≥ 60**	119	81	38	0.262	0.609
**< 60**	84	60	24

**Table 4:** Chi-square test for weight loss and ONS during neoadjuvant chemotherapy

Weight loss	No. of patients (total, *n* = 171)			*χ*^2^	*P*
	Total no. of rows	ONS	No ONS		
**No weight loss**	113	93	20	4.261	0.039
**Weight loss**	90	63	27		

*ONS* oral nutritional supplement

### ROC analysis

**Fig. 2:**
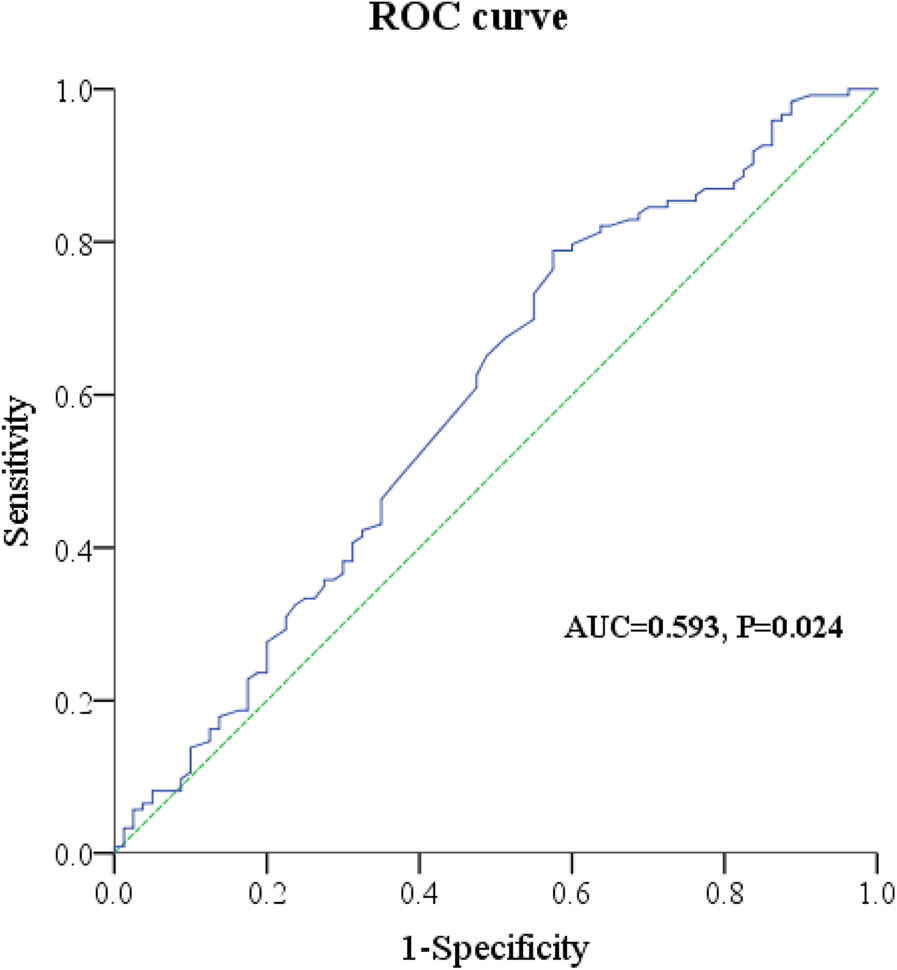
Receiver operating characteristic curve of weight change and neoadjuvant chemotherapy effect. The area under the curve (AUC) was 0.593 (95% CI 0.512–0.675), and the *P* value was 0.024

Since weight loss was an independent risk factor which influenced the effect of neoadjuvant chemotherapy, we tried to figure out the cutoff value of weight change (percentage) during neoadjuvant chemotherapy in order to determine how much weight loss could be more severe. In the ROC analysis (Fig. 2) of weight change and neoadjuvant chemotherapy effect, patients with CAP 0, CAP 1, and CAP 2 were considered chemotherapy effective while patients with CAP 3 were considered chemotherapy noneffective. The area under the curve (AUC) was 0.593 (95% CI 0.591–0.680), and the *P* value was 0.024. The cutoff value of weight change was − 2.95% when Youden Index (sensitivity + specificity − 1) was at its maximum. And the sensitivity was 78.9% while the specificity was 42.5%. The result indicated that weight loss more than 2.95% during the neoadjuvant chemotherapy may bring patients a worse chemotherapy effect.

## Discussion

We sought to assess the association between clinicopathological factors and pathological response of neoadjuvant chemotherapy in a cohort of patients with GC at a medical center. We found that patients < 60 years old, histological type of poor differentiation or signet-ring cell carcinoma, and weight loss during neoadjuvant chemotherapy were independent risk factors which influenced the effect of neoadjuvant chemotherapy.

In our study, we found that patients with well-differentiated or moderately differentiated adenocarcinoma had better pathological response than poorly differentiated adenocarcinoma or signet-ring cell carcinoma. To our knowledge, so far there was little direct evidence to show the relationship between histological type and neoadjuvant chemotherapy sensitivity; two studies [16, 17] found that GC patients with poor histological type showed worse prognosis and survival. Notably, it was consistent with our current findings and in one of the studies [16]; most patients received chemotherapy treatment, which favored our speculation that poor histological type was related to poor chemotherapy effect.

As for age, we found that older patients (≥ 60 years old) had a significant higher rate of pathological response than younger patients (< 60 years old). Lu et al. [16] declared that younger age was associated with poor histological type and worse prognosis. Based on this thesis about age and histological type, we combined age with histological type in order to find out how age affected the effect of chemotherapy in one of our analyses (Table 3). Although the histological type showed no significant difference between young patients and old patients, we still speculated the histological type of younger patients was relatively poorer based on our clinical experience. And poorer histological type led to poorer chemotherapy effect. We believe this trend will become more pronounced as the number of patients increases.

It has been shown that malnutrition in cancer patients is common, and weight loss is an important manifestation of malnutrition [18, 19]. Chemotherapy can often be associated with severe toxicity [20]. One of the side effects of chemotherapy is gastrointestinal reaction which triggers nutrient deficiency and subsequently causes weight loss. Tan et al. [21] and Palmela et al. [22] found that weight loss could further enhance the chemotherapy toxicity and result in poorer chemotherapy tolerance eventually. When fell into such situations, patients had to receive dose delay, dose reduction, even treatment termination. And not surprisingly, these patients had a high chance for poor prognosis. In our study, although there was no significant difference about weight loss in univariate analysis, we found patients with weight loss had worse pathological response than patients without weight loss in multivariate analysis. This meant weight loss did reduce the effect of neoadjuvant chemotherapy.

One of the important ways to avoid weight loss during chemotherapy is oral nutritional supplement (ONS). de van der Schueren et al. [23] found that ONS, especially those enriched with protein and n-3 polyunsaturated fatty acids (PUFA), showed an overall benefit of interventions on body weight during chemotherapy. Similarly in our study, we found that patients without weight loss during neoadjuvant chemotherapy had a higher rate of ONS than patients with weight loss (Table 4). In our ROC analysis, we further found that patients with weight loss > 2.95% during neoadjuvant chemotherapy showed a worse chemotherapy effect, which was consistent with a previous report [24] that weight loss > 2.4% might indicate a worse survival. Limited by sample size, we did not obtain a high AUC value in our study, but the cutoff value (weight loss > 2.95%) could still have some guiding effects on clinical work. We should perform routine nutritional screening for patients during neoadjuvant chemotherapy especially focusing on weight change. Timely nutritional support such as ONS to maintain the patients’ body weight is good for the result of neoadjuvant chemotherapy.

Our present study is not devoid of limitations. First and most significantly, the retrospective nature of the study design and relatively small sample size limit the ability to draw more accurate conclusions. Secondly, the neoadjuvant chemotherapy regimen involved in our study is only SOX regimen. Other chemotherapy options may have different effects on the final results. Finally, our study included patients from one single academic medical center, and therefore the outcomes may not be generalizable. Nevertheless, to the best of our knowledge, this study is the first to clarify which clinicopathological factors can influence the pathological response of neoadjuvant chemotherapy in GC patients, and we innovatively use CAP classification to represent pathological response and chemotherapy effect. Future investigations are needed with prospective, multi-center designs and larger sample sizes to verify the relationship between clinicopathological factors and pathological response of neoadjuvant chemotherapy in GC patients.

## Conclusions

We found that patients < 60 years old, histological type of poor differentiation or signet-ring cell carcinoma, and weight loss during neoadjuvant chemotherapy were independent risk factors for neoadjuvant chemotherapy effect in GC patients. Patients with weight loss > 2.95% during neoadjuvant may have a worse chemotherapy effect. Timely nutritional support such as ONS to maintain patients’ body weight is crucial for improving the effect of neoadjuvant chemotherapy.

## Data Availability

The datasets used and analyzed during the current study are available from the corresponding author on reasonable request.

## Abbreviations

GC: Gastric cancer
ROC: Receiver operating characteristic curve
OR: Odds ratio
CI: Confidence interval
AUC: Area under the curve
ONS: Oral nutritional supplement
IARC: International Agency for Research on Cancer
WHO: World Health Organization
CT: Computed tomography
EUS: Endoscopic ultrasonography
AJCC: American Joint Committee on Cancer
SRCC: Signet-ring cell carcinoma
NCCN: National Comprehensive Cancer Network
ESMO: European Society for Medical Oncology
SOX: Oxaliplatin plus S-1
CAP: College of American Pathologists
SPSS: Statistical Package for the Social Sciences
cT stage: Clinical T stage
cN stage: Clinical N stage
